# Prevalence of Rhinitis in Athletes: Systematic Review

**DOI:** 10.1155/2017/8098426

**Published:** 2017-08-09

**Authors:** Pavol Surda, Abigail Walker, Matus Putala, Pavel Siarnik

**Affiliations:** ^1^Department of Otorhinolaryngology, Guy's and St Thomas' University Hospital, London, UK; ^2^Department of Physical Education and Sports, Comenius University, Bratislava, Slovakia; ^3^Department of Neurology, Faculty of Medicine, Comenius University, Bratislava, Slovakia

## Abstract

**Background:**

Prevalence of rhinitis in athletes has frequently been studied and varies widely from 27% to 74%. The aim of this systematic review was to examine the prevalence of rhinitis in athletes, to specifically compare the evidence of rhinitis in land-based and aquatic athletes.

**Methods:**

Systematic search of MEDLINE, EMBASE, and the non-MEDLINE subset of PubMed was performed from inception to March 8, 2016, to identify studies on rhinitis in athletes.

**Results:**

Of the 373 identified unique articles, a total of 13 studies satisfied the criteria for this review. The final group contained 9 cohort and 4 case-control studies. We found 10 studies that reported the prevalence of allergic rhinitis (21%–56.5%). In contrast, nonallergic rhinitis was identified by only 1 author (6%). We have also evaluated the prevalence of rhinitis in the separate subgroups (land, water, and cold air) where swimmers seem to be the most affected (40%–74%), followed by cross-country skiers (46%) and track and field athletes (21 to 49%).

**Conclusion:**

We did not reveal any convincing trend of a higher prevalence in land-based athletes compared to general population. By contrast, aquatic and cold air athletes demonstrate increased prevalence reflecting the irritant effects of their environment on the nasal mucosa.

## 1. Introduction

Peak sporting performance requires optimal levels of health and fitness. Rhinitis, with its proven detrimental effects on sleep and mood [1], and its association with asthma [2], has clear potential for compromising athletic ability.

The acute effects of exercise on the nose have been well delineated—vasoconstriction of the capacitance vessels results in a measurable increase in nasal volume [3]. In aerobic exercise, nasal minute ventilation increases absolutely but proportionately contributes less than at rest, as the low resistance oral airway is used preferentially [4]. The impact on nasal physiology of repeated exercise training is less well established. Many of the environments and endeavours in which athletes are immersed have the potential for harm to the nasal mucosa. For example, an exercise which takes place in cold air (skiers, snowboarders, and ice hockey) or in chlorinated water (swimmers, divers, and water polo) subjects the nasal mucosa to local irritants. Aerobic exercise which takes place outdoors may result in inhalation of above average volumes of aeroallergens, nitrous oxide, or pollution due to the increased minute ventilation required to sustain activity [5, 6].

Rhinitis in athletes has frequently been studied in combination with asthma, and although there are many literature estimates of prevalence in the athletic population, the estimates of frequency of rhinitis vary widely—from 27% to 74% [7, 8]—which may in part be attributable to differences in the studied populations (e.g., swimmers and runners), the aetiology of the rhinitis studied (irritative, allergic, or undefined), or the diagnostic criteria used.

The reported prevalence of AR in normal population differs from country to country. In a study using the ARIA definition on the European population, the prevalence was found to be around 25% and ranged from 17% to 28.5% [9]. Moreover, an increasing trend in prevalence of AR has been observed over the last decades of the past millennium but in the last 25 years this trend seems to be tailed off [10, 11]. The prevalence of NAR in the normal population is not as well studied as AR but NAR has been reported to account for from 17 to 52% of all cases of rhinitis in adults [12, 13].

The aim of this systematic review was to examine the prevalence of rhinitis in athletes, to specifically compare the evidence of rhinitis in land-based and aquatic athletes.

## 2. Methods

We sought to investigate the prevalence of rhinitis in athletes. The inclusion criteria were as follows: study must evaluate only human study subjects who reached age of 12 years, contain abstract, and be published in the English language between January 1980 and October 2015. We defined an athlete as a person who trains more than 6 hours/week. We classified sports into three categories which represent their different environments: land, water, and cold air.

Our review followed the PRISMA (Preferred Reporting Items for Systematic Reviews and Meta-Analysis) guidelines for reporting. P.S. and A.W. performed a systematic search of MEDLINE (OVID), EMBASE (OVID), and the non-MEDLINE subset of PubMed from inception to March 8, 2016, to identify studies on rhinitis in athletes. Both controlled vocabulary (including MeSH-terms) and words in title, abstract, and author keywords were searched. We excluded studies indexed with animals, but not indexed with humans, conference abstracts and case reports, and studies with trauma or concussion in the title. The search consisted of two concepts: athletes (including all kinds of athletic sports, swimming, and high intensity training) and rhinitis (consisting of synonyms for rhinitis and symptoms and underlying mechanisms of and tests for rhinitis). We cross-checked the reference lists and the citing articles of the identified relevant papers and adapted the search in case of additional relevant studies. The bibliographic records retrieved were imported and deduplicated using ENDNOTE.

Studies meeting the inclusion criteria were assessed on quality using the PRISMA/AMSTAR checklist as a guideline. The studies which did not discuss the subject were excluded. No minimum was set on the number of study subjects.

### 2.1. Data Collection

All abstracts and full-text articles were reviewed by two researchers (P.S. and A.W.) using the following inclusion criteria: cohort designs (case report, case series, other noncohort study designs, and nonsystematic reviews were excluded).

Information obtained from each article included authors, year of publication, number of participants, number of participants per category (land/water/cold air), study design, outcomes, and prevalence. Findings were tabulated and descriptively analysed, listing outcomes measured.

According to the ARIA document, allergic rhinitis is clinically defined as a symptomatic disorder of the nose, induced after allergen exposure due to an immunoglobulin IgE-mediated inflammation. Symptoms of allergic rhinitis include rhinorrhea, nasal obstruction, nasal itching, and sneezing which are reversible spontaneously or with treatment [25]. Based on duration of symptoms, ARIA subdivides AR into intermittent or persistent. NAR is defined as symptoms of rhinitis without positive skin prick test [26].

In this review, we studied the overall prevalence of rhinitis regardless of the phenotype and also AR and NAR separately. The methods used for diagnosis of rhinitis are markedly heterogeneous and in surveys studying athletes poorly reported. Therefore, we reviewed the literature to find the most suitable definition for rhinitis in athletes.

We included the studies with the diagnosis of rhinitis (both nonallergic (NAR) and allergic rhinitis (AR)) based on the “self-reported physician diagnosis/physician diagnosis” or positive answers in a questionnaire. In the case of AR, this had to be accompanied with at least one positive skin prick test result/positive serum specific IgE-test.

## 3. Results

**Figure 1:**
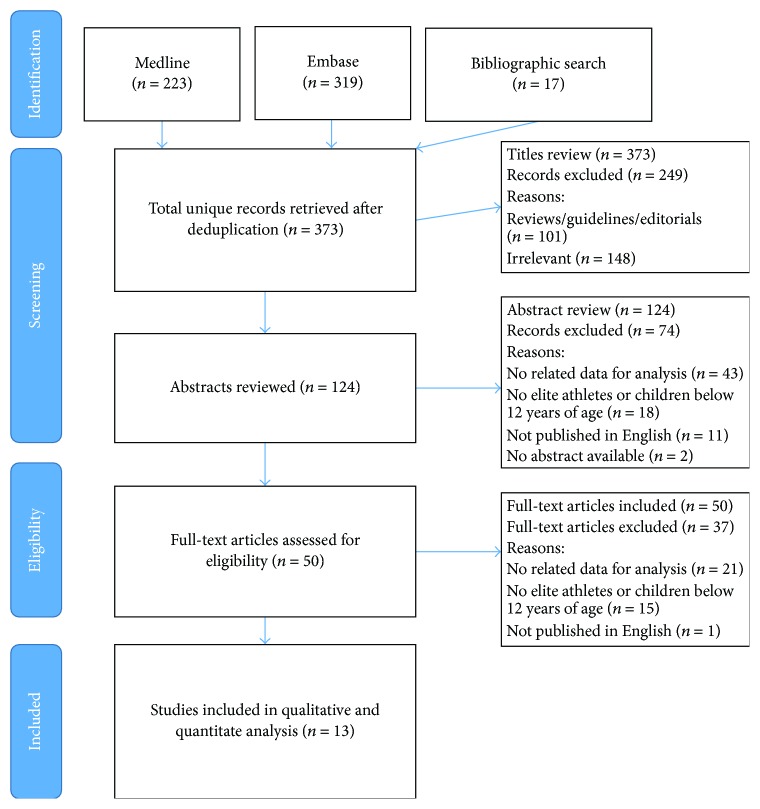
Data extraction and analysis.

A systematic review of titles, abstracts, and full-text publications was performed as described in Figure 1. Of the 373 identified unique articles, a total of 13 studies satisfied the criteria for this review. The characteristics are illustrated in Table 1. Demographic details and efforts to control confounding were incompletely reported. Nine cohort and 4 case-control studies were included. Five authors used for diagnosis the questionnaire with or without SPT/positive serum specific IgE-test and 8 self-reported physician diagnosis/physician diagnosis. A quantitative meta-analysis was not performed due to the heterogeneity of the outcome data.

We found 10 studies that reported the prevalence of AR in which the prevalence ranged from 21% to 56.5%. In contrast, NAR was identified by only 1 author (prevalence 6%) [7].

We have also evaluated the prevalence in the separate subgroups (land, water, and cold air). Rhinitis in swimmers was studied by 3 authors who reported prevalences ranging from 40% to 74% [8, 21, 22]. Rong et al. reported prevalence of physician-diagnosed AR in 56.5% [21]. Two authors focused on the prevalence of overall rhinitis (AR + NAR) which was reported as 40% and 74% [8, 22]. We identified only one study which distinguished the specific prevalence for athletes training in cold environment, reporting rhinitis in 46% [15]. Using track and field athletes to represent the land subgroup does not reveal an increased incidence of AR when compared with the general population (21 to 49%) [7, 17].

## 4. Discussion

This systematic review of the literature demonstrates that the analysis of the prevalence of rhinitis in athletes is hindered by a relatively wide range of reported results and heterogeneity of methods used for diagnosis which are in surveys studying athletes poorly reported.

Lack of consensus evidence and critical reviews on this topic led us to perform this systematic review to examine the prevalence of rhinitis in athletes. Unfortunately, a quantitative meta-analysis was not possible to perform due to the heterogeneity of the outcome data.

As illustrated in Table 1, athletes as a whole population do not seem to suffer with AR more often than general population. The prevalence of AR ranged widely between 21% and 56.5%. NAR, although evaluated by only 1 author, was found to be 6% (Table 1). The true prevalence of NAR in normal population is not known, and precise data are difficult to obtain as NAR can coexist with allergic rhinitis, and therefore the comparison between athletes and normal population is difficult to perform [12, 27]. Noticeable is also the stable prevalence of AR in athletes over the last 25 years which is comparable with the normal population [10, 11]. It is unfortunate that we had to exclude studies dated from the last decades of the past millennium due to poorly reported diagnostic methods, and therefore a long-term comparison with the normal population cannot be achieved.

The wide range of results might also suggest heterogeneity of either the population or sampling methods. Therefore, we have examined the prevalence in the separate subgroups (land, water, and cold air) according to the environment where they spent the most training hours. After doing so, the range narrows and is easier to interpret. For example, track and field athletes do not suffer from rhinitis significantly more than general population, regardless of being endurance or sprint specialists [18]. On the contrary, 48.6% of athletes who spend training hours in cold environment report rhinitis and the distinctive symptom is rhinorrhea (96%), often severe [15]. Aquatic environment causes a similar pattern, with analysis of all studied swimmers reporting rhinitis in 56% to 74% [8, 21, 28]. Bougault et al. in the case-control study showed a significant difference of rhinitis prevalence (AR + NAR) between the swimmers and healthy controls (74% versus 40%, resp., *P* < 0.01) [8]. The assumption would be that the rhinitis in swimmers is nonallergic in nature due to chlorine irritation and coexists with AR probably in higher prevalence compared to the normal population. Unfortunately, studies which satisfied the inclusion criteria did not examine the prevalence of NAR phenotype separately. Athletes are also known to suffer with exercise-induced rhinitis which is characterized by short-term rhinitic symptoms triggered by the exercise [29]. Interestingly, the prevalence of exercise-induced rhinitis was similar in between swimmers and runners [30]. We have excluded studies examining this entity as our study was focused on chronic rhinitis.

Moreover, many patients with rhinitis without asthma demonstrate nonspecific bronchial hyperresponsiveness after exercise or methacholine, this being a risk factor for developing asthma which is reported between 3.7% and 22.8% in athletes. If epidemiological studies indicate that rhinitis and asthma often coexist, prospective studies suggest that rhinitis frequently precedes the development of asthma [28]. When the two risk factors, sporting activity and atopy, were combined in a logistic regression model, the relative risk of asthma was surprisingly high: 25-fold in an atopic speed and power athlete, 42-fold in an atopic long-distance runner, and 97-fold in atopic swimmers compared with nonatopic controls [31].

## 5. Conclusion

Rhinitis in the athlete is an emerging field of interest. The published evidence identified in this systematic review illustrates the need for precision when analysing this particular population: to take care to define both the individual studied (and their exercise environment) and the condition identified (allergic or nonallergic). Although individual studies suggest an increase in prevalence of rhinitis in the land-based athlete, systematic review of the literature does not reveal any convincing trend of a higher prevalence compared to the general population. By contrast, aquatic and cold air athletes both demonstrate an increased prevalence of rhinitis—reflecting the irritant effects of their environment on the nasal mucosa.

## Figures and Tables

**Table 1:** Prevalence of Rhinitis in Athletes.

Study	Design	Participants (*n*)	Number of participants per environment	Diagnosis	AR	NAR	Rhinitis (AR + NAR)
Katelaris et al. (2000) [14]	Cohort study	214	96 L, 118 W, 0 CA	Quest. + SPT	41%		

Bonadonna et al. (2001) [15]	Cohort study	144	0 L, 0 W, 46 CA	Self-reported physician diagnosis			46%

Alaranta et al. (2005) [16]	Case-control study	446 + 1504 controls	266 L + W, 180 CA	Self-reported physician diagnosis	36.1%		

Silvers and Poole (2006) [17]	Cohort study	55	55 L, 0 W, 0 CA	Self-reported physician diagnosis	49%		

Katelaris et al. (2006) [18]	Cohort study	980	980 W + L, 0 CA	Quest. + SPT	24%		

Macucci et al. (2007) [19]	Cohort study	352	352 L, 0 W, 0 CA	Quest. + SPT	22.16%		

Bonini et al. (2007) [20]	Case-control study	98	98 W + L, 0 CA	Quest. + SPT	34.7%		

Rong et al. (2008) [21]	Case-control study	23 + 30 controls	0 L, 23 W, 0 CA	Self-reported physician diagnosis	56.5%		

Clearie et al. (2010) [22]	Cohort study	61	61 W	Physician diagnosis			40%

Thomas et al. (2010) [23]	Cohort study	291	291 L + W, 0 CA	Self-reported physician diagnosis	25.4%		

Bougault et al. (2010) [8]	Case-control study	39 + 30 controls	0 L, 39 W, 0 CA	Physician diagnosis + SPT			74%

Kurowski et al. (2016) [7]	Cohort study	222	214 L, 8 W, 0 CA	Physician diagnosis + SPT	21%	6%	27%

Bonini et al. (2015) [24]	Cohort study	659	606 L, 38 W, 15 CA	Quest. + SPT	26.2%		

L: land, W: water, and CA: cold air.
